# Institutional delivery service utilization and associated factors in fragile and conflict-affected situations in Sekota town, Northern Ethiopia, 2022: A community-based cross-sectional study

**DOI:** 10.1016/j.heliyon.2023.e16239

**Published:** 2023-05-12

**Authors:** Getachew Muluye Gedef, Abeba Gashaw, Desalegn Anmut Bitew, Fantahun Andualem

**Affiliations:** a School of Midwifery, College of Medicine and Health Science, University of Gondar, Gondar, Ethiopia; b Department of Maternal and Child Health, Amdework Primary Hospital, Waghimra Zone, Amhara Region, Ethiopia; c Department of Reproductive Health, Institute of Public Health, College of Medicine and Health Science, University of Gondar, Gondar, Ethiopia; d Department of Psychiatry, College of Medicine and Health Science, University of Gondar, Gondar, Ethiopia

**Keywords:** Institutional delivery, Fragile and conflict, Northern Ethiopia

## Abstract

**Background:**

Conflict-affected areas are considered to contribute a substantial proportion of worldwide maternal deaths. However, research on maternal health care in conflict-affected countries is very limited. In the absence of recent data, it is impossible to monitor progress made in mitigating the effect of conflict on maternal survival. As a result, this study targeted to assess institutional delivery services usage and influencing factors in a fragile and conflict-affected situation in Sekota town, Northern Ethiopia.

**Methods:**

A community-based cross-sectional study was employed among 420 mothers in Sekota town, Northern Ethiopia from July 15th to 30th, 2022. The desired sample size was determined using a single population proportion formula. The data were collected by using interviewer administered structured questionnaire; entered via EpiData version 4.6 and analyzed using SPSS version 25 software. To identify the associated factors, a bivariable and multivariable logistic regression model was applied. The level of significance was declared at a p-value of <0.05. An adjusted odds ratio with a 95% confidence interval was considered to see the strength of the association between dependent and independent variables.

**Results:**

Of the total respondent, 202 (48.1%), 95% CI: (43.0%, 53.0%) mothers utilized institutional delivery service. The use of institutional delivery services was associated with the maternal educational level of secondary school and above (AOR = 2.06, 95% CI: 1.08–3.93), antenatal care during the most recent pregnancy (AOR = 5.24, 95% CI: 3.01–9.11), being informed on birth preparedness and complication readiness (AOR = 1.93, 95% CI: 1.23–3.02) and displacement of the respondents from their usual place of residence due to conflict (AOR = 0.41, 95% CI: 0.21–0.68).

**Conclusion:**

Institutional delivery service utilization was very low in the study setting. Healthcare for women in conflict-prone areas requires critical attention and should be given priority during the conflict. More prospective research is needed to fully understand and reduce the impact of conflict on maternal and neonatal health care.

## List of abbreviations and acronyms

ANC: Antenatal care
AOR: Adjusted odds ratio
BPCR: Birth preparedness and complication readiness
CI: Confidence interval
COR: Crude odds ratio
EDD: Expected date of delivery
FCAS: Fragile and conflict-affected situation
MHCS: Maternal health care services
MNH: Maternal and neonatal health

## 1 Introduction

Fragile and conflict-affected situations (FCAS) have substatial impacts on the health of affected community [1,2]. The threat may be in the form of direct harm or disruption of the provision and use of health services, particularly it hinders access to maternal and reproductive health services which results in unfavorable maternal and newborn health outcomes [3,4]. According to global statistics, 60% of preventable maternal deaths which often can be prevented with the usage of skilled birth attendants and 45% of neonatal deaths take place in FCAS where political unrest, displacement or forced migration, and natural disasters prevail [1].

FCAS is a common problem in low and middle-income countries; findings highlight that low utilization of maternal and neonatal health (MNH) services in FCAS and resulting in poor MNH status [5]. Sub-Saharan Africa (SSA) alone accounted for 201,000 of the estimated 303,000 maternal mortalities worldwide in 2015, and the majority of these are attributed to complications of pregnancy and childbirth due to lack of institutional delivery by skilled attendants and emergency obstetric care [5,6].

According to the 2016 Ethiopian Demographic Health Survey (EDHS) maternal mortality in Ethiopia is high, with 412 deaths per 100,000 live births [7]. If the women had access to maternal health care services like institutional delivery this death could have been prevented. Due to the conflict that broke out in Northern Ethiopia in November 2020 between the Ethiopian federal government on one side and the Tigray People's Liberation Front (TPLF) on the other, the health facility was destroyed and many people were left without access to basic medical care. There have also been numerous instances of sexual and gender-based violence against women and girls.

Although FCAS has a negative impacts on health and health systems, rigorous research on the indirect impacts of conflict and its effect on health inequalities, particularly for women and children, has not been adequately and quantitatively investigated [8]. Evidence is particularly sparse on MNH in acute crisis and remains limited in fragile situations generally. The qualitative analysis tried to identify some factors influencing service provision like insecurity, unavailability of the workforce, lack of funds, and inconsistent supplies [8]; the availability of healthcare providers was negatively affected by the protracted nature of the conflict and insecurity in the region [9].

Reducing the global maternal mortality ratio to less than 70 per 100,000 live births by 2030 is one of the targets of the Sustainable development goal (SDG). To achieve this goal strong and effective strategies as well as accurate measurement and monitoring of progress on key indicators are crucial. More context-specific research and collaboration are needed to close the gap in women's reproductive health, a gap further widened in FCAS. FCAS in SSA needs special attention as their maternal health indicators are suggested to be exceedingly poor and their progress lags globally.

In Ethiopia besides the importance of institutional delivery, there was very limited evidence regarding the raised issues and no study was done in the study setting. Therefore, this study addresses this gap by assessing the level of institutional delivery service utilization and associated factors in fragile and conflict-affected situations in Sekota town, the Northern part of Ethiopia. The results of this study will raise awareness of the effects of armed conflicts on the accessibility and usage of maternal health services in affected communities, draw attention to the concerned body, and provide information for formulating and implementing policies and strategies that can reduce the risk associated with conflict in similar settings. They will also serve as a baseline for future research.

## 2 Materials and methods

### 2.1 Study design, setting and period

A community-based cross-sectional study was carried out in Sekota town from July 15 to 30, 2022. Sekota is the capital town of the Waghimra zone, Amhara Regional State, Ethiopia and it is located around 720 km from Addis Ababa, the capital city of Ethiopia, and 540 km northeast of Bahir Dar, the capital of the Amhara region. Administratively, Sekota town has four kebeles, and Based on the 2012 census conducted by the central statics agency of Ethiopia this town has a total population of 27,459 of whom 14,237 are female. In the town, there is one governmental general hospital, one health center, four health posts, and three privately owned clinics. The healthcare facilities provide maternity services in addition to other medical services.

### 2.2 Populations and the eligibility criteria

All women who had given birth in the past two years preceding the data collection period and residents of the town were the study population whereas women who were currently displaced from other areas, acutely mentally ill, and unwilling to participate were excluded from the study.

### 2.3 Sample size and sampling procedure

A single population proportion formula, n = (Zα/2)^2^ P (1-P)/d^2^, was used to calculate the sample size with the following assumptions. Using p = 46.2% prevalence of institutional delivery service in a prior study conducted in west Ethiopia [6], with a 95% Confidence Interval (CI) and 5% marginal error and considering a 10% non-response rate. Finally, it gave a sample size of 420.

There are a total of four kebeles (Kebele 1, Kebele 2, Kebele 3, and Kebele 4) in the town and all of the kebeles were considered in the study. The number of households found in the area was obtained at the kebeles' health posts, and a sampling frame was created using that information. The desired sample size was then proportionally distributed according to the size of the households in each kebeles. The simple random sampling technique was used to select the study participants. When there were multiple (more than one) eligible women living in a household, a woman was chosen by lottery method for the interview, and for houses without an eligible woman, the next-closest household was contacted.

## 3 Data collection tools and procedures

Data were gathered using interviewer-administered structured and pretested questionnaires. The questionnaire was designed after a review of works of literature on the same subject (modified standardized questionnaires) and it comprises information related to conflict situations, maternal socio-demographic conditions, obstetric and gynecologic history, and current pregnancy characteristics. Data were collected by four data collectors who had previous experience with data collection. Two supervisors were in charge of supervising the data collection procedure.

### 3.1 Assurance and data quality control

The questionnaire was written originally in English and then translated into Amharic, which is the language spoken locally in the study setting. Then, to ensure consistency, it was translated back into English by linguists. A pre-test was conducted on 42 participants (10% of the sample size) in a nearby town (Woldia town) to determine the appropriateness of the instrument before the actual data were collected. The necessary measure was then taken. Inappropriate questions and unclear wording were removed and replaced. Some variables like residency and distance from the health facilities were excluded from the tool since the sample was obtained from a fixed geographical catchment area. The data collectors and supervisors received a one-day training from the principal investigator on the general aim of the study, sampling techniques, and data collection methods. The data was checked before and after the data entry stage to ensure its consistency & completeness.

### 3.2 Data processing and analysis

The collected data were entered into Epi-Data version 4.6 and exported to SPSS version 25 software for analysis. The results were organized, compiled, and presented using descriptive statistics. Bivariable and multivariable logistic regression models were used to identify the associated factors for the use of institutional delivery services. The level of significance was declared at a p-value of less than 0.05 for both steps. An adjusted odd ratio with a 95% CI was considered to measure the strength and direction of the association between the dependent and independent variables. We conducted Hosmer Lemeshow tests to check the model's goodness of fit. Multicollinearity was checked using the Variance inflation factor (VIF), in which a VIF of less than ten was considered to be acceptable.

### 3.3 Study variables

Institutional delivery service utilization was the outcome variable. The independent variables were **conflict-related factors**: the destruction of health facilities, the irregular opening of health facilities (inconvenient time of services), looting or disruption of medical supplies, fleeing of health care providers, disrespectful approach and lack of privacy, women's displacement away from the health facility, movement restriction to operational health facility due to insecurity; **socio-demographic factors**: age, ethnicity, marital status, educational level, occupation, husband educational level, source of information on maternal health care services (MHCS) and autonomy on self-health care decision and **obstetrics and gynecologic related factors**: gravidity, parity, history of cesarean delivery, bad obstetrics history, the intention of last pregnancy, antenatal care (ANC), knowledge on the expected date of delivery (EDD) and counseling on birth preparedness and complication readiness (BPCR).

### 3.4 Operational definitions

**Institutional delivery service utilization** is a delivery that takes place at any medical facility staffed by qualified and skilled birth attendants [10]. It was assessed by asking the mother whether or not used the services with “Yes” or “No” responses.

**Fragile situations (Insecure and Unstable)** are defined as periods when states or institutions lack the capacity, accountability, or legitimacy to mediate relations between citizen groups and between citizens and the state, making them vulnerable to violence [11].

**Conflict-affected situations** are characterized as situations that are in or have experienced severely disruptive conflict(s) [11].

**Disrespectful maternity care during childbirth**: Physical abuse, non-consented clinical care, non-confidential care, non-dignified care (including verbal abuse), discrimination of patients, abandonment of care, and detention in facilities by health care professionals during childbirth [12]**.**

**Bad obstetrics history:** any previous unfavorable fetal and maternal outcome.

**Residents of the town** if a woman who lives in the town for six months and above.

### 3.5 Ethics approval and consent to participate

The study was carried out per the ethical standards. It was reviewed and approved by the Institutional Review Board of the University of Gondar and it granted ethical clearance (Reference number: VP/RTT/05/947/2022). The aim of the study and the right of the respondents to decline to participate or withdraw at any time were explained. Before starting the interview, we got everyone's fully informed consent. Anonymity and confidentiality were maintained by excluding personal identifiers from the data collection tool.

## 5 Results

### 5.1 Socio-demographic characteristics of the study participants

In this study, a total of 420 mothers were interviewed, making a response rate of 100%. Out of the interviewed mothers, more than half (52.1%) of them were in the age category of 20–29 years. The mean age of the participants was 29.04 years, with a standard deviation (SD) of 5.04. The vast majority of the participants 373 (88.8%) were Orthodox Christian in religion and regarding their marital status, 404 (96.2%) of respondents were in a relationship. For about 264 (62.9%) of respondents, the health institutions were the main sources of information on maternal health care services (MHCS) (Table 1).

**Table 1:** Socio-demographic characteristics of study participants in Sekota town, July 2022 (n = 420).

Variables	Category	Frequency	Percentage
Age	15–19	16	3.8
	20–29	219	52.1
	30–39	184	43.8
	40–49	1	0.2
Marital status	In a relationship	404	96.2
	Not in a relationship	16	3.8
Ethnicity	Amhara	401	95.5
	Tigray	11	2.6
	Others^a^	8	1.9
Religion	Orthodox	373	88.8
	Muslim	37	8.8
	Others^b^	10	2.4
Education	Cannot read and write	95	22.6
	Can read and write	76	18.1
	Elementary education	134	31.9
	Secondary and above	115	27.4
Occupation	Housewife	222	52.9
	Governmental employee	100	23.8
	Private business	70	16.7
	Others^c^	28	6.7
Husband education (n = 404)	Cannot read and write	111	27.5
	Can read and write	16	3.9
	Elementary education	56	13.9
	Secondary and above	221	54.7
Autonomy on self-health care decision	Yes	293	69.8
	No	127	30.2
Source of Information on MHCS	Health institution	264	62.9
	Social Medias	122	29.0
	Others^d^	34	8.1

**MHCS:** Maternal health care service.

a Oromo, Afar and South.

b Protestant, Catholic.

c Student, Daily laborer.

d Family, Friend and women group association.

### 5.2 Obstetric characteristics of the study participants

Nearly, three-fourths (76.9%) of the total participants had a previous birth experience and of those 41 (12.7%) were delivered by cesarean section. About 290 (69.0%) of the mothers had attended ANC visits during the last pregnancy. Among the mothers who attended ANC, only 124 (42.8%) mothers had ANC contacts of four and above. About 147 (35.0%) of the respondents didn't know their expected date of delivery and 226 (53.8%) of the respondents were not informed about BPCR during pregnancy. Of the total respondents, 202 (48.1%) mothers utilized institutional delivery services with a 95% CI of 43.0%–53.0% (Table 2).

**Table 2:** Obstetrics characteristics of study participants in Sekota town, July 2022 (n = 420).

Variables	Category	Frequency	Percentage
Gravidity	Primigravida	87	20.7
	Multigravida	333	79.3
Parity status	Nulliparous	97	23.1
	Parous	323	76.9
History of previous cesarean delivery (n = 323)	Yes	41	12.7
	No	282	87.3
Bad obstetrics History	Yes	56	13.3
	No	364	86.7
Intended pregnancy	Yes	348	82.9
	No	72	17.1
ANC follow-up in the last pregnancy	Yes	290	69.0
	No	130	31.0
Number of ANC contacts	<4	166	57.2
	≥4	124	42.8
Know the EDD	Yes	273	65.0
	No	147	35.0
Counseled on BPCR	Yes	226	53.8
	No	194	46.2
Place of delivery	Health institution	202	48.1
	Home	218	51.9

**ANC**: Antenatal care; **EDD:** Expected date of delivery; **BPCR**: Birth preparedness and complication readiness.

### 5.3 The fragile and conflict-affected situation related factors

From the total, about 193 (46.0%) of respondents claimed they were treated disrespectfully by a healthcare professional while utilizing the service and 237 (56.4%) of respondents stated that there was movement restriction because of conflict which makes using the institutional delivery service very challenging. In addition, 39.0% of respondents said they had been shifted to a different place (conflict-free zone) that was distant from health facilities (Table 3).

**Table 3:** Fragile and conflict-related factors in Sekota town, July 2022 (n = 420).

Variables	Frequency	Percentage
Think the disrespectful approach of health workers is a problem		
Yes	193	46.0
No	227	54.0
Think lack of privacy is a problem during institutional delivery		
Yes	209	49.8
No	211	50.2
Movement restriction to operational health facilities due to insecurity		
Yes	237	56.4
No	183	43.6
Displacement of the women away from health facilities		
Yes	164	39.0
No	256	61.0
Fleeing of local health providers from the conflict-affected area		
Yes	217	51.7
No	203	48.3
Irregular opening of health facilities (inconvenience)		
Yes	213	50.7
No	207	49.3
Looting or disruption of medical supplies to the health facilities		
Yes	205	48.8
No	215	51.2
Destruction of health facilities or complete shutdown of health facilities		
Yes	265	63.1
No	155	36.9

### 5.4 Factors affecting institutional delivery service utilization

We analyzed different factors for their independent effect on the utilization of institutional delivery services. We implemented binary logistic regression to assess the association of the independent variables with the occurrence of dependent variables. Accordingly, the model showed that statically significant association of utilization of institutional delivery with maternal educational level, source of information, parity, bad obstetrics history, the intention of last pregnancy, ANC, EDD, awareness on PBCR, the approach of health care provider, women's displacement from the conflict-affected area, movement restriction to operational health facility due to insecurity and non-functional (destruction and/or shutdown) of health facilities.

Variables that were found to be associated with the dependent variable in the bivariable analysis (P ≤ 0.05) were taken to the multivariable analysis. After adjusting for possible confounding factors in the multivariable logistic regression, maternal educational level of secondary school and above, attending ANC visits during the last pregnancy, being well aware of BPCR and women's displacement from the conflict-affected area were found to be independent predictors of institutional delivery service utilization.

The odds of using institutional delivery service were two times (AOR = 2.06, 95% CI: 1.08–3.93) greater among mothers with secondary-level education and above than among moms with lower levels of education. Mothers who attended antenatal care visits during the most recent pregnancy had a higher likelihood of using institutional delivery services than mothers who didn't (AOR = 5.24, 95% CI: 3.01–9.11). Similarly, utilizing institutional delivery service was two times highly likely if a mother was counseled on BPCR (AOR = 1.93, 95% CI: 1.23–3.02). Contrarily, mothers who had been forced to leave their usual place of residence due to the conflict were 59% times less likely to seek institutional delivery services (AOR = 0.41, 95% CI: 0.21–0.68) (Table 4).

**Table 4:** Bivariate and multivariable logistic regression analysis of factors associated with institutional delivery service utilization of study participants during fragile and conflict-affected situations in Sekota town, northern Ethiopia, 2022 (n = 420).

Variables	Institutional Delivery		COR (95%CI)	AOR (95% CI)	*P*-Value
	Yes	No			
Educational status					
Secondary and above	61 (30.2)	54 (24.8)	**1.77 (1.02**–**3.07)**	**2.06 (1.08**–**3.93)**	**0.028**
Primary school	66 (32.7)	68 (31.2)	1.52 (0.89–2.59)	1.66 (0.89–3.10)	0.111
Able to read and write	38 (18.8)	38 (17.4)	1.57 (0.85–2.88)	1.69 (0.83–3.42)	0.148
Unable to read and write	37 (18.3)	58 (26.6)	Reference	Reference	
Source of information on MHCS					
Health professional	149 (73.8)	115 (52.8)	**3.11 (1.43**–**6.76)**	2.08 (0.83–5.17)	0.117
Media	43 (21.3)	79 (36.2)	1.30 (0.57–2.98)	0.85 (0.33–2.22)	0.741
Others	10 (5.0)	24 (11.0)	Reference	Reference	
Parity status					
Parous	164 (81.2)	159 (72.9)	1.60 (1.01–2.54)	1.47 (0.85–2.57)	
Nulliparous	38 (18.8)	59 (27.1)	Reference	Reference	0.172
Bad obstetrics history					
Yes	34 (16.8)	22 (10.1)	1.80 (1.01–3.20)	1.86 (0.95–3.63)	0.070
No	168 (83.2)	196 (89.9)	Reference	Reference	
Intended pregnancy					
Yes	175 (86.6)	173 (79.4)	1.68 (1.00–2.84)	1.28 (0.68–2.39)	0.442
No	27 (13.4)	45 (20.6)	Reference	Reference	
ANC follow-up in the last pregnancy					
Yes	178 (88.1)	112 (51.4)	7.01 (4.25–11.59)	5.24 (3.01–9.11)	**0.001**
No	24 (11.9)	106 (48.6)	Reference	Reference	
Knowledge on EDD					
Yes	143 (70.8)	130 (59.6)	1.93 1.30–2.85)	1.01 (0.621.66)	0.970
No	59 (29.2)	88 (40.4)	Reference	Reference	
Counseled on BPCR					
Yes	128 (63.4)	103 (47.2)	1.64 (1.09–2.46)	1.93 (1.23–3.02)	**0.004**
No	74 (36.6)	115 (52.8)	Reference	Reference	
Disrespectful approach from health workers					
Yes	105 (52.0)	88 (40.4)	0.62 (0.42–0.92)	0.74 (0.40–1.34)	0.317
No	97 (48.0)	130 (59.6)	Reference	Reference	
Movement restriction due to insecurity					
Yes	96 (47.5)	141 (64.7)	0.49 (0.33–0.73)	0.84 (0.46–1.55)	0.577
No	106 (52.5)	77 (35.3)	Reference	Reference	
Displacement of the women away from health facilities					
Yes	56 (27.7)	108 (49.5)	0.39 (0.26–0.58)	0.41 (0.21–0.68)	**0.001**
No	146 (72.3)	110 (50.5)	Reference	Reference	
Destruction or shutdown of health facility					
Yes	110 (54.5)	155 (71.1)	0.48 (0.32–0.72)	0.73 (0.41–1.31)	0.292
No	92 (45.5)	63 (28.9)	Reference	Reference	

***AOR:****Adjusted odds ratio;****COR****: Crude odds ratio;****ANC:****Antenatal care;****BPCR:****Birth preparedness and complication readiness;****EDD****: Expected date of delivery;****MHCS:****Maternal health care services*.

## 6 Discussion

This community-based study set out to determine the prevalence of institutional delivery service utilization and associated factors in fragile and conflict-affected situations. Accordingly, the prevalence of institutional delivery service utilization was found to be 48.1% (95% CI: 43.0%–53.0%). This result is nearly similar to those studies done in Chelia District (46.2%) [6] and Northwest Ethiopia (47.3%) [13].

However, the finding of this study is lower than those of a study conducted in the central Gondar zone (58.17%) [14], Delgi district in northwest Ethiopia (75.9%) [15], Bule Hora Town (72%) [16], Ghana (77.89%) [17], Nepal (60.9%) [18]. The disparity in findings may be because the current study takes into account the situational factors when the utilization of institutional delivery service is less likely. This result is also supported by a study done in Cameroon which found that the utilization of deliveries attended by qualified personnel dropped from 46% in 2017 to 26% in 2018 during the armed conflict [2]. Moreover, studies conducted in Egypt, Afghanistan, and Pakistan, as well as multi-country research in FCAS, found that access to crucial reproductive and maternal health services was significantly lower in conflict zones than in non-conflict ones [4,8,19,20]. This is due to the fact that conflicts may prevent women from using delivery services in a variety of ways, including by reducing the availability of maternal and reproductive health care services by destroying health facilities; causing a shortage of medical professionals because they might have left hospitals to avoid risk; endangering local security and women tend to become less likely to acquire institutional delivery [21].

The current study finding is slightly higher than a study done in Gura Dhamole Woreda (29.2%) [22] and Mandura district (38%) [23]. The possible reasons for the discrepancy in prevalence may be the time gap (year of the study) and sample size between the present study and the previous studies. Over time, the community's awareness of maternal health care services has grown so that level of utilization of the services gets improved.

The odds of using institutional delivery service were two times greater among mothers with secondary-level education and above than among moms with lower levels of education. This finding is consistent with reports of previous studies done in Northwest Ethiopia [15], Bule Hora town [16], Papua New Guinea [24], and Nepal [18]. The possible explanation is due to the fact that women with a higher level of education are more likely to be conscious of their health and more likely to understand the need for institutional delivery and skilled birth attendant use.

Mothers who attended ANC visits during the most recent pregnancy had a higher likelihood of using institutional delivery services than mothers who didn't. This finding is in line with a study conducted in Ethiopia [25], Papua New Guinea [24], Ghana [17], and Nepal [18]. Similarly, utilizing institutional delivery service was two times highly likely if a mother was counseled on BPCR. This conclusion is supported by earlier research that showed mothers who have a discussion with their spouse and health extension workers about the place of delivery during a home visit, and experience with or knowledge of pregnancy danger signs are more likely to utilize institutional delivery services [6,13,16,22].

Contrarily, mothers who had been forced to leave their usual place of residence due to the conflict were 59% less likely to seek institutional delivery services. The Armed conflicts have the potential to compromise security, drive women away from healthcare facilities, reduce household income, and deter them from seeking out institutional birth. In conclusion, armed conflicts may worsen maternal and child health by restricting women's access to prenatal and delivery care, increasing the risk of pregnancy-related morbidity for mothers and the likelihood of adverse birth outcomes. So during and after conflict, greater healthcare efforts are needed to improve the quality and coverage of maternal healthcare to enhance maternal survival as well as protect women and children from the indirect harms of conflict are very crucial.

### 6.1 Strength and limitations of the study

The current study is the first to investigate situational factors that affect institutional delivery service utilization in the study area. This study uses data from a primary source and is conducted at the community level so that the findings of the study can be generalized to all women in the study area. However, being cross-sectional, it is difficult to conclude the causes or temporal correlations between the studied variables. Due to various constraints, all potentially contributing variables (influencing factors) were not investigated. Since our estimation sample only includes mothers who survived, our estimates are probably lower than the actual consequences of conflict. A further drawback to the study is the potential for recall bias and social desirability among the respondents.

## 7 Conclusions

The utilization of institutional delivery service in a fragile and conflict-affected situation in the study area was 48.1%, with 95% CI: 43.0%–53.0%. Respondents' displacement from their usual place of residence due to the conflict to a non-conflict affected zone was negatively associated with the institutional delivery service utilization. Whereas attending antenatal follow-up visits, being informed on birth preparedness and complication readiness and a maternal higher level of education were positively associated factors of institutional delivery service utilization. Therefore, healthcare for women should be prioritized during acute and protracted periods of conflict. By enhancing community-based health services in the camp and establishing mobile clinics and outreach programs, healthcare professionals must update their maternal health strategy in conflict-affected areas. The impact of conflict on maternal and neonatal health care requires additional prospective research.

## Authors' contribution

Each of the aforementioned authors made a significant contribution to the work and gave their approval for publishing. G.M made contributions to the conception and design, acquisition, analysis, and interpretation of data, as well as the preparation of the original manuscript. A.G contributed to data entry, analysis, and interpretation of the data. F.A made contributions to the conception, design, analysis, and revision of the document. D.A.B revised the document critically. All authors read and approved the final version to be published.

## Availability of data and materials

The data sets used and or analyzed during the current study are available from the corresponding author on reasonable request via email.

## Funding statement

No specific funding was received for this work.

## Declaration of competing interest

The authors declare that they have no known competing financial interests or personal relationships that could have appeared to influence the work reported in this paper.
